# The Local Application of Salicylates

**Published:** 1910-08-06

**Authors:** W. Essex Wynter

**Affiliations:** Physician to the Middlesex Hospital.


### Hospital Clinics.

### THE LOCAL APPLICATION OF SALICYLATES.

(Abstract of a Lecture delivered at the Polyclinic, July 11, 1910.)

By W. Essex Wynter, M.D., B.S., F.R.C.P., F.R.C.S., Physician to the Middlesex Hospital.

Dr. Wynter offered some suggestions to show that the local application of soluble salicylate—more particularly the salicylate of methyl—might be more efficacious, especially in local rheumatic disorders, than the more usual form of administration by the mouth.

As an instance he cited the case of two youths now in the Middlesex Hospital who developed signs of pericarditis while convalescing from an attack of rheumatic polyarthritis and still taking salicylate of soda, which subsided in three days under the application of salicylate of methyl to the præcordia. In connection with pericarditis he wished to draw attention to a very important indication which seemed to have been hitherto overlooked, as it was not mentioned in the published accounts of the disease. That was loss of abdominal respiratory movement. The association was so constant that, on the one hand, this sign should always lead to examination of the pericardium, even when there was nothing else to call attention to morbid state of that sac; and on the other hand, when such respiratory movement was definitely present, doubtful indications of pericarditis might be disregarded. A very striking instance of this connection between loss of abdominal movement and pericarditis was furnished in the case of a friend's butler who was seized with acute epigastric pain while waiting at dinner. Absence of abdominal movement was so conspicuous that the man was thought to have a perforated gastric ulcer. Other signs being absent and the pain diminishing with rest in bed, this view was abandoned, and on the following day, feeling much better, the man got up and went out of his own accord. The pain, however, returned with increased severity, and on further examination a pericardial rub was obvious on the third day. He was admitted to hospital, and made a good recovery under treatment by salicylate of methyl locally, but for quite a fortnight there was no return of abdominal movement.

### The Effect in Cases of Pericarditis.

Experience of these and other cases showed that important secondary lesions resulted from suspended action of the diaphragm, for it was not a definite paralysis. First among them was loss of function of the lower lobes of the lungs with some degree of collapse, respiration being carried on entirely by the upper lobes, by movements of the front of the chest. Then there was passive dilatation of the stomach, and to a less extent of other hollow viscera, and finally difficulty in evacuation of the bowel, owing to the embarrassment caused to respiration by sustained contraction of the abdominal muscles when the diaphragm was yielding and ready to mount into the thorax. These effects were not merely inferred, but demonstrable by means of the *x*-ray screen, which showed the diaphragm to be stationary in a raised position, and also enabled the upward movement of the kidneys, and even of opaque masses in the intestine, with each inspiration, to be seen.

These remarks, important as their bearing was on the recognition of a serious and obscure disorder, were somewhat of a digression, for the object of mentioning pericarditis was to place it first in the list of disorders satisfactorily treated by the local application of salicylates. The actual plan adopted was to spread an ointment composed of gaultherium oil ʒii. in an ounce of lanoline on lint and apply it continuously over the præcordia. That the medicament was rapidly absorbed was proved by the detection of salicylates in the urine within half an hour. No local irritation was produced, and the only inconvenience resulted from the rather penetrating odour and the presence of grease on the chest interfering with examination. But these were trifles compared with the benefit accruing, and were easily averted by changing the dressing.

It is hardly necessary to state that this mode of treatment was particularly applicable to rheumatic pericarditis, which was, however, by far the commonest. Rapid subsidence of the inflammation probably exercised an important influence in diminishing the formation of adhesions and extension to the fibrous sac and myocardium, and so limited the later serious consequences of pericarditis. Where effusion occurred the fluid rapidly disappeared under similar local treatment, and it might be of interest to record that in such cases the loss of abdominal respiratory movement, though obvious, was not so complete as in plastic exudation, and examination with the *x*-ray screen showed such effusion clearly, the outline being distinguished from that of a dilated heart by absence of contractile oscillation.

Further examination of these cases showed that some effusion sometimes existed at the sides and back of the heart, where no corresponding signs were afforded by the ordinary means of clinical observation.

### Some Other Indications.

As a sequel to ordinary rheumatic polyarthritis, it was not unusual to find pain with stiffness and even swelling persisting in some one joint, after the general fever had subsided. In such cases the local application of salicylates appeared often more efficacious than the continued administration by the mouth, and offered the advantage that the curative effect could be thus maintained, without causing the nausea, prostration, and deafness which were liable to attend the former when unduly prolonged.

Many cases of sore throat—faucial injection and follicular, or parenchymatous tonsilitis commonly attributed to rheumatism—responded rapidly to a compress extending to the angle of the jaw and composed either of the ointment of gaultherium or of a hot fomentation to which a drachm of the oil had been added.

Perhaps of all the disorders affected by this application none showed such marked improvement as erythema nodosum. In the ordinary way the blotches subsided spontaneously in the course of two, four, or six weeks, being unaffected by remedies and prolonged by repeated outbreaks. The local application of methyl salicylate with menthol as an ointment relieved the discomfort at once and induced immediate subsidence within a week, no fresh blotches appearing. One curious feature during convalescence was the tendency of the patches to turn temporarily a blue colour after the patient had been up for a while, as though paralytic dilatation of the capillaries permitted the stasis of venous blood, which subsided in a short while after resuming the horizontal position.

The widest application, however, of this mode of treatment occurred in the various aspects of fibrositis due to sub-acute rheumatism affecting nerves, muscles, aponeuroses, and bursæ. Sciatica, brachial neuritis, facial paralysis, and other varieties of local trouble due to rheumatic changes in nerve sheaths usually responded rapidly to the continuous application of the ointment in severe cases, or its intermittent inunction in mild ones, without much local tenderness. Muscular rheumatism, stiff neck, pleurodynia, lumbago, and teno-synovitis, mostly in young people, disappeared rapidly under the influence of one of the liquid preparations.

Bursitis was often a rather puzzling complaint, causing pain and tenderness, which were not referred actually to a joint, though often in close proximity; the neighbourhood of the shoulder, hip, elbow, knee, or tuber ischii being common sites. One of the least known was the middle of the calf, where a large bursa existed between the soleus and gastrocnemius, which was liable to inflammation from over-exertion or exposure to wet and cold.

The useful influence of salicylate was not, however, restricted to rheumatism, for, as sodium salicylate internally was of service in acute stages of gout in reducing fever and pain, so the local application of methyl salicylate was one of the best curative agents for acute gout, particularly in one part, such as the foot; a hot fomentation containing a drachm of gaultherium oil, or one of its preparations with menthol, was the best thing to apply.

After enumerating these many conditions in which the local application of salicylates was of service, it only remained to mention the preparations which were available.

The stock remedy was an ointment of two drachms olei gaultherii in an ounce of lanoline, with the addition of 15 grains of menthol where an anæsthetic effect was required. Antiphlogistine served as a useful poultice, containing sufficient methyl salicylate to be effective.

A mixture of methyl salicylate with menthol made into a thin ointment was sold in collapsible tubes as anæsthol, a convenient remedy for inunction in mild affections, especially chilblains.

The liquid preparations were mesotan, kasemol, and betul oil.

Kasemol, a vinegar-like fluid, was one of the least objectionable in regard to odour and absence of grease; mesotan was similar, but irritating, and best used as a liniment with an equal quantity of olive oil. Betul oil was nearly pure methyl salicylate.

